# Crosstalk between autophagy and metabolic regulation of (CAR) T cells: therapeutic implications

**DOI:** 10.3389/fimmu.2023.1212695

**Published:** 2023-08-22

**Authors:** Ahmad Reza Panahi Meymandi, Behnia Akbari, Tahereh Soltantoyeh, Jamshid Hadjati, Daniel J. Klionsky, Behnam Badie, Hamid Reza Mirzaei

**Affiliations:** ^1^ Department of Medical Immunology, School of Medicine, Tehran University of Medical Sciences, Tehran, Iran; ^2^ Life Sciences Institute and Department of Molecular, Cellular, and Developmental Biology, University of Michigan, Ann Arbor, MI, United States; ^3^ Division of Neurosurgery, City of Hope Beckman Research Institute, Duarte, California, United States; ^4^ Department of Genetics, University of Pennsylvania Perelman School of Medicine, Philadelphia, PA, United States; ^5^ Institute for Immunology and Immune Health, University of Pennsylvania Perelman School of Medicine, Philadelphia, PA, United States

**Keywords:** CAR T cell, metabolism, autophagy, tumor microenvironment, adoptive cellular therapy (ACT)

## Abstract

Despite chimeric antigen receptor (CAR) T cell therapy’s extraordinary success in subsets of B-cell lymphoma and leukemia, various barriers restrict its application in solid tumors. This has prompted investigating new approaches for producing CAR T cells with superior therapeutic potential. Emerging insights into the barriers to CAR T cell clinical success indicate that autophagy shapes the immune response via reprogramming cellular metabolism and vice versa. Autophagy, a self-cannibalization process that includes destroying and recycling intracellular components in the lysosome, influences T cell biology, including development, survival, memory formation, and cellular metabolism. In this review, we will emphasize the critical role of autophagy in regulating and rewiring metabolic circuits in CAR T cells, as well as how the metabolic status of CAR T cells and the tumor microenvironment (TME) alter autophagy regulation in CAR T cells to restore functional competence in CAR Ts traversing solid TMEs.

## 1 Introduction

In recent years, chimeric antigen receptor (CAR) T cell therapy has emerged as a groundbreaking approach for treating advanced hematological malignancies, revolutionizing cancer immunotherapy (1). CAR T cells are designed to recognize tumor-associated antigens on the surface of cancer cells and initiate intracellular signaling pathways that activate T cells, promote their proliferation, and enable the killing of tumor cells. The approval of the first anti-CD19 CAR T cell therapy by the US Food and Drug Administration (FDA) in 2017 marked a significant milestone in the field (2, 3). However, despite the success of CAR T cell therapy, several challenges remain, including toxicity, limited efficacy in solid tumors, reduced persistence, and inadequate tumor infiltration (4). To overcome these limitations, researchers are exploring novel strategies to engineer CAR T cells with enhanced anti-tumor activity. Among these approaches, metabolic regulation of CAR T cells has emerged as a promising avenue to reprogram these cells, making them more resistant to cytotoxicity and exhaustion while promoting their persistence.

*Autophagy*, an evolutionarily conserved self-destructive cellular process, is an active mechanism that occurs during metabolic deprivation (5). The term “autophagy” originates from the Greek words meaning “eating of self.” During autophagy, cellular cargo is delivered to lysosomes, where it is degraded and recycled, either selectively or non-selectively. This process is crucial for maintaining energy balance within cells (6). Autophagy is triggered under conditions of physiological stress, such as amino acid starvation, and serves as a survival mechanism by recycling cytoplasmic macromolecules. Among the three main types of autophagy (microautophagy, macroautophagy, and chaperone-mediated autophagy), macroautophagy is particularly relevant to T cell metabolism. Therefore, this review will primarily focus on macroautophagy. Macroautophagy, hereafter referred to as autophagy, is the most common form of autophagy observed in cells. It involves the formation of transient double-membrane structures, known as phagophores, around cytoplasmic components, including organelles, aggregated proteins, or microbes. Phagophores develop into autophagosomes, which subsequently fuse with lysosomes for the degradation and recycling of their contents (7). Autophagy plays a critical role in regulating various aspects of T cell biology, including T cell development, survival, and the formation of memory T (T_mem_) cells (8). Recently, the role of autophagy in modulating T cell fate and function, mainly by influencing T cell metabolism, has gained increasing attention. T cells undergo metabolic reprogramming upon activation to meet their energy requirements. Autophagy serves as a link between T cell signaling and metabolic processes in response to T cell receptor (TCR) and interleukin 2 (IL-2) stimulation. In this review, we provide an overview of the intricate interplay between autophagy and metabolism in CAR T cells. We also discuss potential strategies for targeting autophagy and metabolism to engineer metabolically fit CAR T cells with enhanced anti-tumor potential.

## 2 Overview of molecular mechanisms of autophagy in T Cells

**Figure 1:**
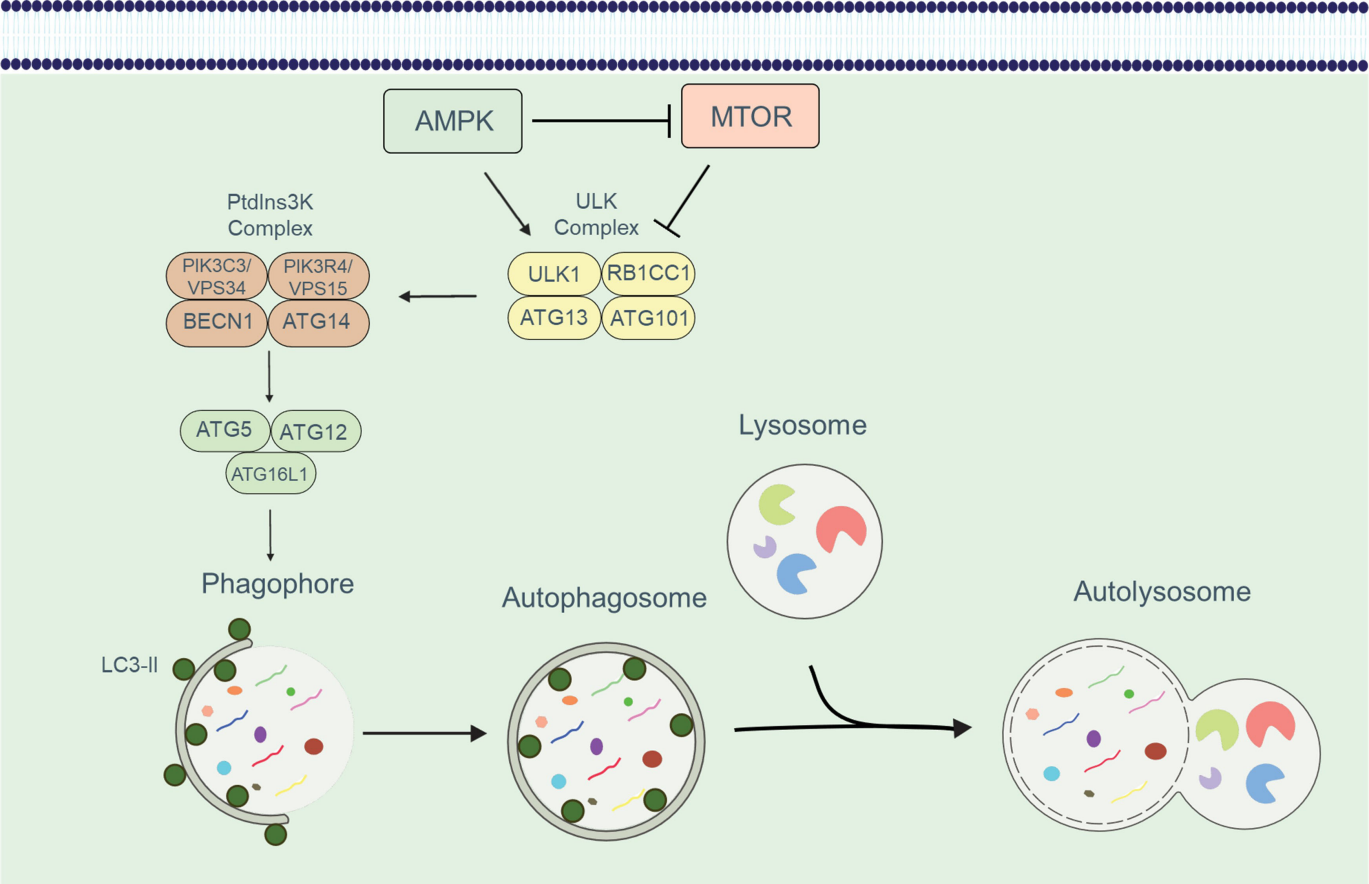
An overview of the autophagic process. The development of the phagophore and the recruitment of the ATG16L1 complex is induced by the sequential activation of the ULK and PtdIns3K class III complexes. The ATG16L1 complex along with LC3-II enables the phagophore to expand and form an autophagosome. Autophagosome formation and cargo isolation are aided by the LC3/Atg8 conjugation system. Following isolation, the cargo is degraded in an autolysosome, which forms when autophagosomes and lysosomes fuse.

Autophagy has emerged as a crucial regulatory mechanism in the immune system, significantly influencing various aspects of T cell function. This cellular pathway governs the fate of different T cell subsets, and its activity is modulated by several factors and cellular complexes. Autophagy is activated in response to diverse scenarios, such as the engulfment and elimination of intracellular proteins, organelles, and pathogens through the formation of autophagic membrane structures (9). The molecular machinery involved in autophagy comprises a series of proteins, many of which are designated as ATG (autophagy-related) proteins. The initiation, expansion, and maturation of phagophores, the initial structures of autophagy, are tightly regulated. The ULK1/Atg1 complex plays a pivotal role in forming the phagophore structure (10). This complex, consisting of ULK1/ULK2, RB1CC1/FIP200, ATG13, and ATG101, activates the BECN1 (beclin 1, autophagy-related)-PIK3C3/VPS34 phosphatidylinositol 3-kinase (PtdIns3K) class III complex, which generates phosphatidylinositol-3-phosphate (PtdIns3P). The recruitment of PtdIns3P-binding molecules is integral to the process of phagophore formation (11). Two ubiquitin-like conjugation systems are essential for autophagy: the ATG12–ATG5-ATG16L1 system and the ATG8-family proteins, which include MAP1LC3/LC3 or GABARAP (in this review, we refer to LC3 for simplicity) (12). These conjugation systems facilitate the lipidation of LC3 and the subsequent formation of autophagosomes. During LC3 lipidation, LC3 is proteolytically processed from its precursor form, LC3-I, to the lipidated form, LC3-II, which functions as a docking site for cargo receptors on phagophore membranes (13). The cargo receptors connect the autophagy machinery to specific cargoes, and subsequently, the phagophore expands and seals, giving rise to the autophagosome (14). The autophagosome then fuses with the lysosome, forming an autolysosome (15). Within the autolysosome, the cargoes are degraded by lysosomal enzymes, and the breakdown products are released through permeases for recycling (16). This process is shown in **Figure 1**.

## 3 Role of autophagy in T cell immunobiology

**Figure 2:**
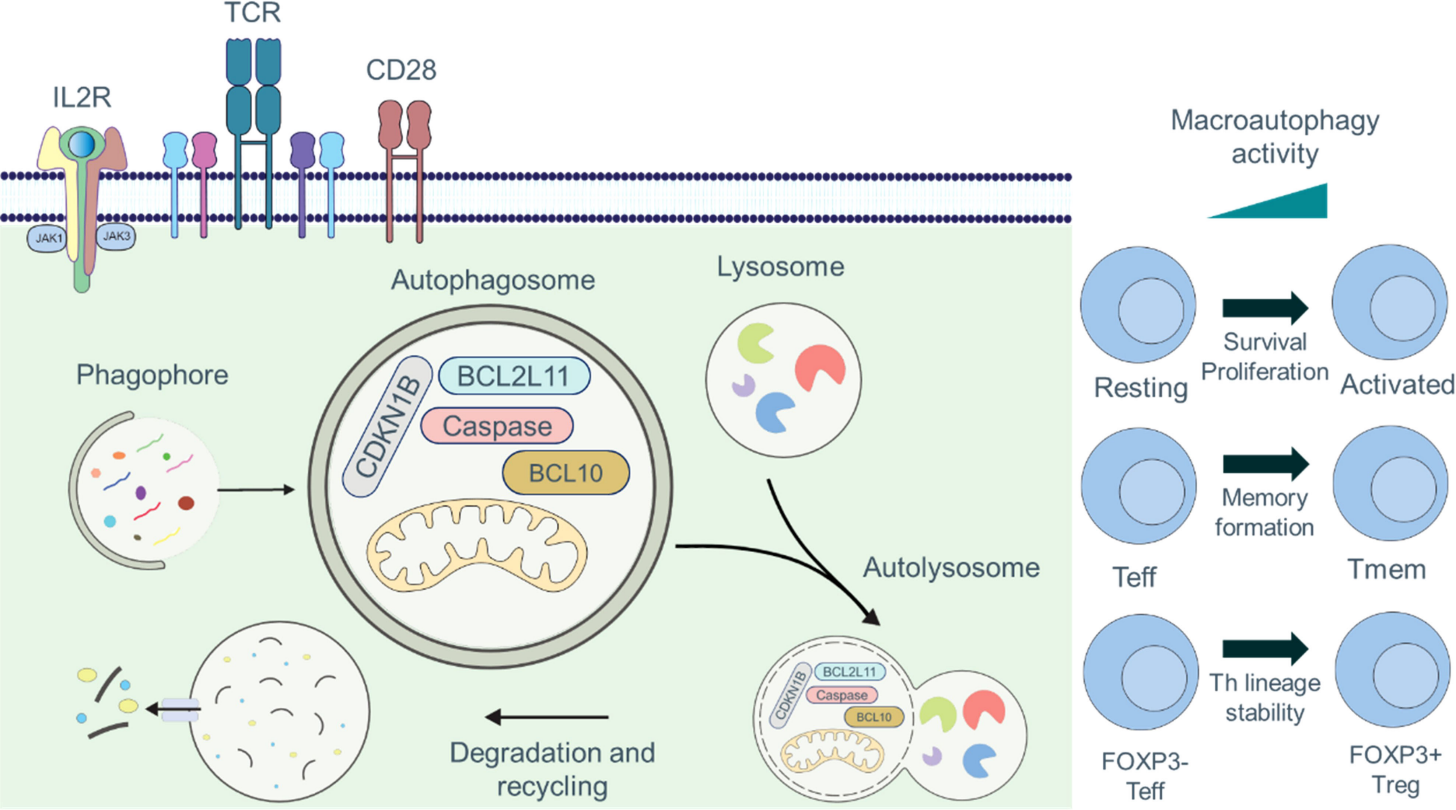
Roles of autophagy in T cell fate and function. Engagement of the TCR and CD28 signaling pathways is required for full activation of T cells, which can be further augmented by signaling through IL2R. Autophagy is maintained at basal levels in naïve and resting T cells, and the cargo of autophagosomes includes organelles, such as mitochondria, thus acting as an important quality control mechanism. Autophagy is induced by TCR- and IL2R-signaling. During activation, the cargo composition changes to primarily cytosolic components, and activation-induced degradation of certain proteins (CDKN1B, caspases, BCL2L11/Bim, or BCL10) has been described to regulate proliferation, survival, and activation. Autophagy plays crucial functions in controlling T cell metabolism, recycling cellular basic components, TCR signal transduction, and breaking down macromolecules to produce signaling metabolites. Additionally, macroautophagy is essential for T cell activation, cell survival, proliferation, and the production of CD8^+^ T_mem_ cells.

Autophagy is critical in various aspects of T cell immunobiology, including T cell survival, proliferation, TCR signaling, and T cell memory formation (**Figure 2**). In this section, we elaborate on the vital role of autophagy in T cell immunobiology.

### 3.1 T cell survival

Previous studies have shown that prolonged persistence of T cells is associated with durable remission in patients with cancers, marking T cell survival as one of the key players in adoptive cellular therapy. Autophagy is essential for T cell survival, and its activity increases significantly upon T cell activation (17, 18). Autophagy-deficient T cell models have provided insights into the homeostatic role of autophagy in T cell proliferation and survival (19–21). Studies using T cell-specific Atg3, Atg5, and Atg7-deficient mice have demonstrated increased apoptosis rates and higher expression of CASP9 (caspase 9) (19, 20, 22). Similarly, BECN1/beclin-1-deficient T cell models exhibit elevated apoptosis rates with increased levels of proCASP3, CASP8, BCL2, and BCL2L11/Bim (23). Autophagy, particularly mitophagy, also regulates mitochondrial turnover as a quality control mechanism to maintain mitochondrial homeostasis (20, 22). Autophagy-deficient T cells show increased mitochondrial and endoplasmic reticulum content, leading to T cell death due to accumulation of reactive oxygen species (ROS) resulting from impaired removal of damaged organelles (19, 20, 22, 24). As a result of autophagy-related changes in mitochondrial turnover and pro-apoptotic factors, autophagy-deficient models exhibit reduced numbers of CD4+ and CD8+ T cells.

### 3.2 T cell proliferation

Besides defects in survival, defects in proliferation may also explain reduced CD4^+^ and CD8^+^ T cell numbers in autophagy-deficient cells, as autophagy regulates T cell proliferation (19, 25). T lymphocytes deficient in Atg5 and Atg7 fail to efficiently proliferate upon TCR stimulation (18, 19). Autophagy-deficient T cells are unable to enter the S phase after TCR stimulation. This defect is attributed to the accumulation of CDKN1B/p27, a negative T cell cycle regulator that is usually degraded by autophagy. Genetic deletion of a single *Cdkn1b* allele restores proliferation, indicating that autophagy regulates T cell proliferation mainly through selective degradation of CDKN1B (25). In summary, autophagy is essential for degrading organelles, apoptotic proteins, and cell cycle regulators, thereby ensuring proper T cell proliferation and survival.

### 3.3 TCR signaling

Following TCR stimulation, many downstream signaling events are required to initiate and maintain activation, and at least one major TCR signaling event is modulated by autophagy. BCL10, a key mediator of TCR-to-NFKB/NFκB signaling, is recognized by the autophagic cargo receptor SQSTM1/p62 and degraded upon effector T (T_eff_) cell activation. Inhibition of autophagy leads to the accumulation of BCL10, suggesting that autophagy prevents excessive TCR signaling, which could result in inappropriate T cell responses (26). In addition, autophagy-mediated degradation of PTPN1 removes its inhibitory effects on downstream signaling pathways of the TCR. Consequently, impaired autophagy results in PTPN1 accumulation, leading to reduced T cell priming response and diminished subsequent stimulation response (27).

### 3.4 T cell memory formation

Aside from regulating T cell proliferation, survival, and TCR signaling, autophagy regulates CD8+ T cell memory formation (28, 29). *Atg7*-deficient CD8^+^ T cells in mice show impaired memory function (28, 29). Studies have also found that defects in CD8^+^ T cell memory formation are related to failure to induce metabolic switches, such as upregulation of fatty acid oxidation, which is essential for CD8^+^ T cell memory formation (28–30). Consequently, these studies shed light on the role of autophagy in modulating T cell homeostasis.

## 4 Metabolic signature in T cell immunobiology

**Figure 3:**
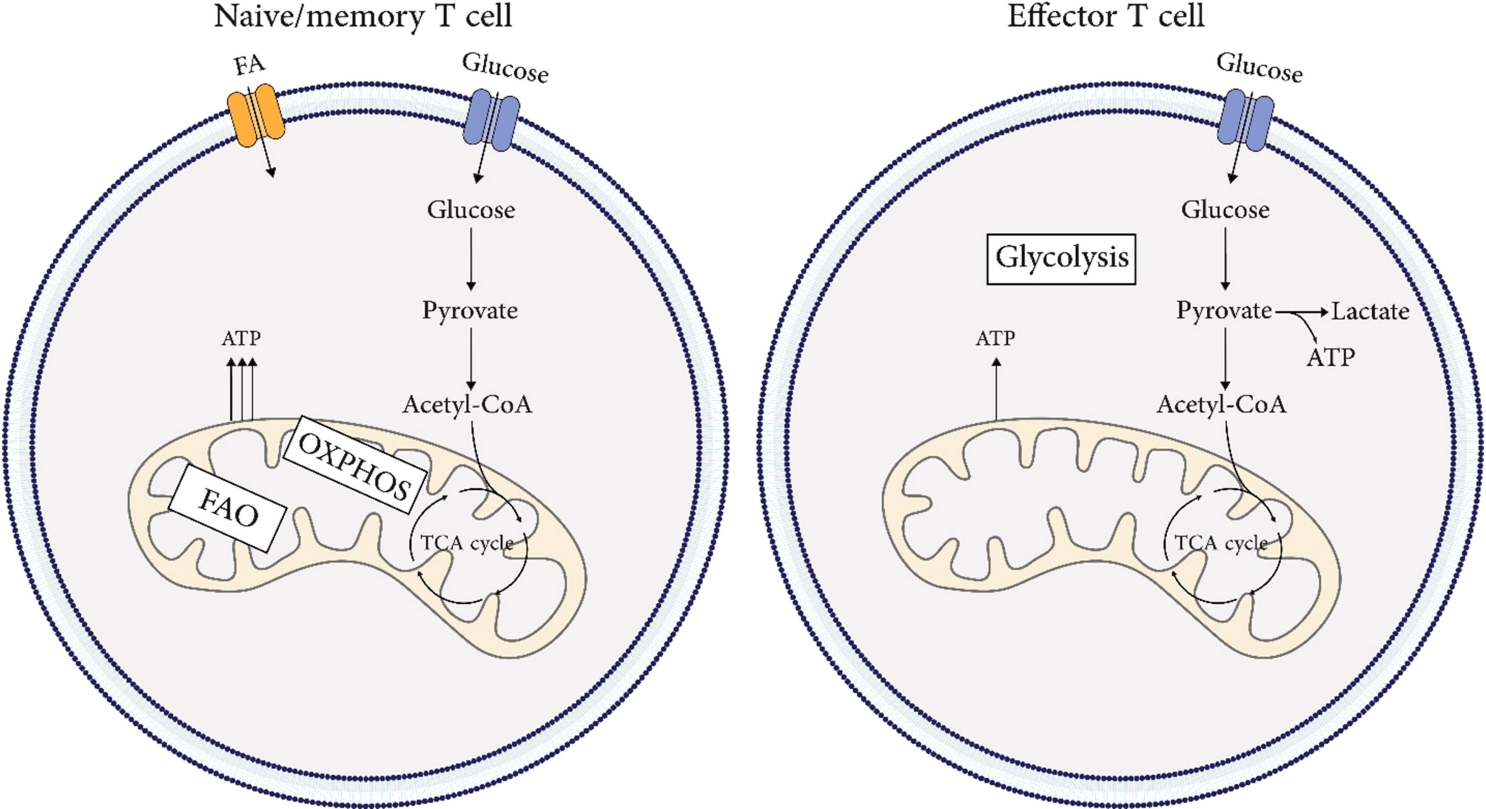
Metabolic signature in T cells immunobiology. Metabolism of naïve and, memory T cells depends on oxidative phosphorylation (OXPHOS) and fatty acid oxidation (FAO) to provide energy. The metabolic pattern of memory T cells is similar to that of naïve T cells, but oxidative phosphorylation is slightly higher, which allows them to be quickly activated after encountering an antigen. The switch from resting naïve or memory T cells to highly proliferative effector T cells needs extensive metabolic reprogramming. As mitochondrial oxidative phosphorylation and ROS rise, aerobic glycolysis is also induced rapidly.

Metabolism of naïve T cells depends on oxidative phosphorylation (OXPHOS) and fatty acid oxidation (FAO) to provide energy. The switch from resting naïve T cells to highly proliferative T_eff_ cells needs extensive metabolic reprogramming. As mitochondrial oxidative phosphorylation and ROS rise, aerobic glycolysis is also induced rapidly (31–33). Upon antigen recognition, extensive metabolic reprogramming occurs. Activation of the PI3K-AKT-mTOR pathway leads to the activation of transcription factors such as HIF1A/HIF-1α and MYC/c-Myc, which in turn upregulate the expression of SLC2A1/GLUT1 (solute carrier family 2 member 1) to promote glycolysis (34). This metabolic switch is known as the “Warburg effect,” where glycolysis is enhanced even in the presence of sufficient oxygen (35). In aerobic glycolysis, glucose is metabolized into pyruvate and converted into lactate independently of mitochondria. This allows the naïve T (T_n_) cell to meet its metabolic needs for rapid proliferation and cytokine production to differentiate into T_eff_ cells. The metabolic pattern of T_mem_ cells is similar to that of T_n_ cells, but oxidative phosphorylation is slightly higher, which allows them to be quickly activated after encountering an antigen (36). Unlike T_eff_ cells, T_mem_ cells do not rely mainly on aerobic glycolysis but prefer OXPHOS. This process is fueled partially by mitochondrial catabolism of intracellular fatty acids (36, 37). Additionally, T_mem_ cells have increased mitochondrial mass and spare respiratory capacity, providing metabolic advantages for surviving and recalling after exposure to antigen (31, 36). **Figure 3** illustrates the metabolic signature in T cell immunobiology.

## 5 Crosstalk between autophagy and metabolism in (CAR) T Cells

**Figure 4:**
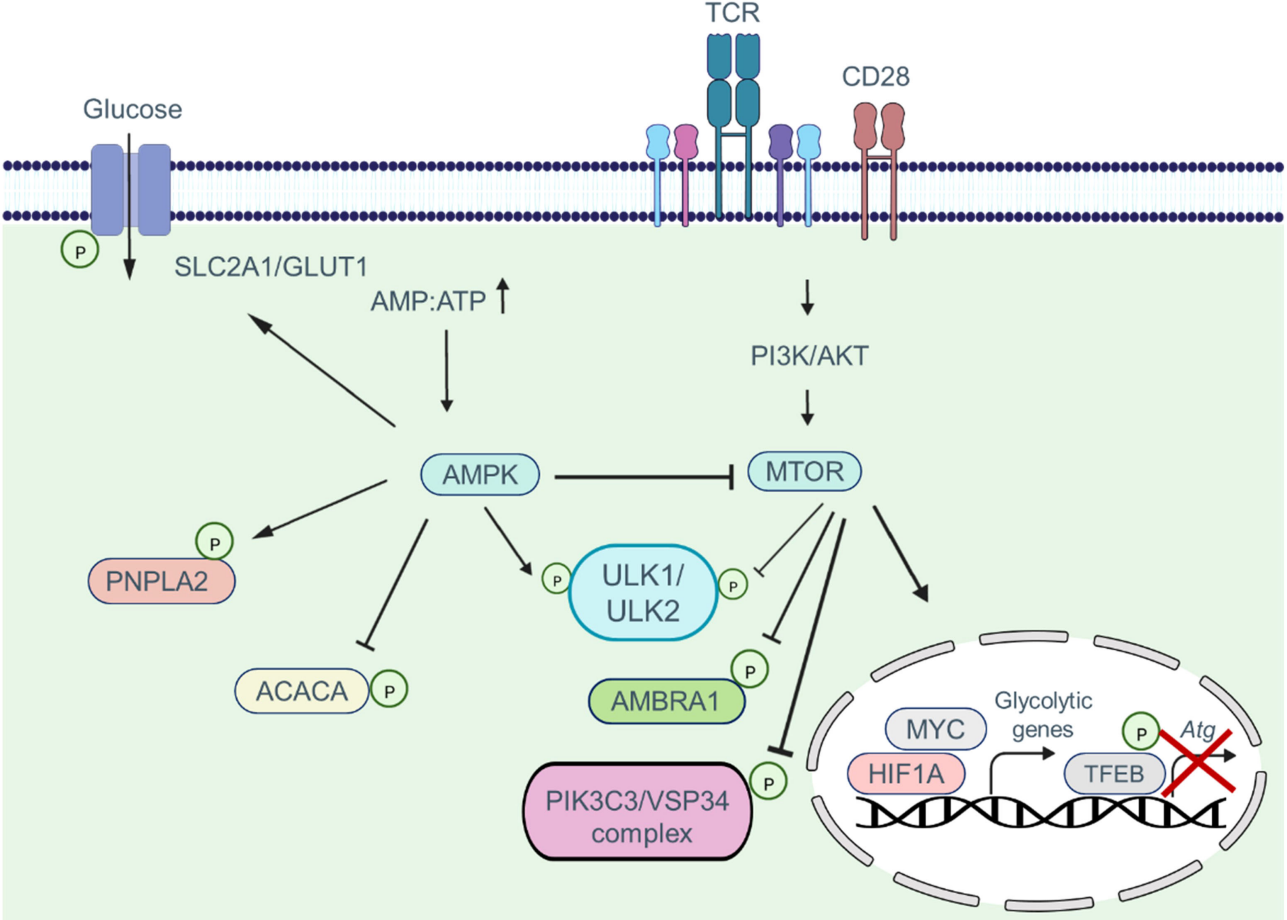
The combination of MTOR and AMPK signaling pathways coordinate and regulate metabolic pathways via the autophagic activity of T cells. MTOR activation suppresses autophagy by phosphorylating autophagy pathway components such as the ULK1/ULK2 complex, AMBRA1, and the PIK3C3/VPS34 complex. Moreover, MTOR signaling promotes phosphorylation and deactivation of the transcription factor TFEB, which is involved in the transcription of *Atg* genes. In addition, when the MTOR pathway is activated, the transcription factors MYC and HIF1A enter the nucleus and induce the transcription of glycolytic genes. AMPK activation, in contrast, inhibits the MTOR pathway and induces phosphorylation of autophagy pathway components, which in turn enhances the autophagic activity within cells. Furthermore, AMPK shifts metabolism toward OXPHOS phosphorylating and activating the SLC2A1/GLUT1 channel, as well as the PNPLA2/ATGL and ACACA/ACC1 enzymes.

Common nutrient-sensing pathways integrate autophagy and metabolism in CAR T cells. The MTOR (mechanistic target of rapamycin kinase) and AMP-activated protein kinase (AMPK) signaling pathways work together to coordinate metabolic pathways and autophagic activity based on environmental conditions, nutrient availability, and energy status (**Figure 4**) (38–40). During cellular differentiation, both metabolism and autophagy need to accurately sense nutrient levels and respond accordingly to optimize nutrient utilization, storage, and recycling (41, 42). MTOR complex 1 (MTORC1) plays a critical role in sensing various stimuli, including growth factors and nutrients. The PI3K-AKT signaling axis promotes anabolism by activating MTOR networks, which modulate metabolic landscapes through transcriptional reprogramming (43). Transcription factors such as HIF1A, SREBF, and MYC/c-Myc are involved in this reprogramming process (34, 44).

Energy depletion triggers the activation of AMPK. When the ATP : AMP ratio decreases, AMP allosterically stabilizes and activates AMPK by binding to its phosphorylated form (45). AMPK directly phosphorylates metabolic enzymes and transporters, including PNPLA2/ATGL, SLC2A1/GLUT1, and ACACA/ACC1, to regulate energy balance (46–48). MTORC1 and AMPK control autophagy by regulating ULK1 kinase. In nutrient-rich conditions, MTORC1 phosphorylates ULK1 and ULK2, inhibiting autophagy (38). MTORC1 also phosphorylates AMBRA1, inhibiting the activity of the PIK3C3/VSP34 complex and influencing autophagy transcriptional regulation through TFEB phosphorylation and repression (49, 50). However, when nutrients are limited, the inhibitory effect of MTORC1 on ULK1/ULK2 is alleviated. Decreasing the ATP : AMP ratio activates AMPK, leading to the phosphorylation and activation of ULK1/ULK2 (51). ULK1/ULK2, in turn, promotes autophagy and may play a crucial role in providing alternative nutrient sources during metabolic stress (38). The interplay between T cell function, metabolism, and autophagy suggests their close association and highlights the importance of autophagy in regulating T cell metabolism, which subsequently impacts T cell function.

### 5.1 Autophagy signature governing (CAR) T cell metabolism

Autophagy can influence metabolic pathways (52), and the autophagic pathway plays a crucial role in successful metabolic reprogramming during immune cell differentiation. In recent years, studies have demonstrated the active role of cellular metabolism in determining T cell fate and function (53, 54). Additionally, autophagy is an essential catabolic process in T cells, and it can modulate the differentiation and function of CAR T cells by regulating metabolism. T_eff_ cells deficient in autophagy produce less ATP during activation, exhibit decreased glycolysis and mitochondrial respiration, and show impaired cytokine secretion and cell proliferation (18). Autophagy degrades proteins, lipids, and glycogen, providing energy substrates and inhibiting this catabolic process can hinder efficient T cell activation (55). Recent research suggests that autophagy can regulate T cell metabolism by activating MTORC1. TAX1BP1 regulates autophagy, where autophagy-mediated degradation of cytosolic proteins provides L-cysteine to activate MTORC1 and induce metabolic changes (56). CD4+ T cells lacking TAX1BP1 fail to initiate the glycolytic anabolic changes necessary for meeting the energy demands of activation, resulting in reduced expression of HK2 (Hexokinase 2) and SLC2A1/GLUT1 (56).

Autophagy is crucial for meeting the metabolic demands of CAR T cell activation and regulating T cell metabolism during CD8+ T_eff_ to T_mem_ differentiation. The presence of central memory T cells (T_cm_) and stem cell-like memory T cells is vital for the success of CAR T cell therapy (57). Several studies have demonstrated the connection between autophagy and memory T cell differentiation, survival, and function (29, 58, 59). T cells lacking Atg5 shift significantly toward an effector memory phenotype and produce higher levels of IFN-γ and TNF-α (59). Mechanistically, Atg5^-/-^CD8+ T cells exhibit increased glucose metabolism, resulting in changes in histone methylation, increased H3K4me3 density, and transcriptional activation of metabolic and effector target genes (59). Autophagy-deficient CD8+ T cells show defects in memory formation due to disrupted metabolism, particularly in mitochondrial FAO (29). Unbiased metabolite data reveals dysregulated mitochondrial FAO, critical for memory T cell formation (30), in Atg5 or Atg7-deficient T cells at the peak of clonal expansion, precisely when antigen-specific T cells transition to the memory phase (29). Autophagy’s ability to degrade lipid droplets also contributes to the switch from glycolysis to FAO required for efficient CD8+ memory formation (60). Autophagy-deficient T cells may also exhibit impaired mitochondrial turnover, leading to other metabolic defects (22). A study by Yang et al. discovered that *pik3c3/vps34* deletion reduces mitochondrial activity during T cell activation (61). The precise utilization and generation of substrates for mitochondrial FAO by memory T cells, and the direct regulation of mitochondrial FAO by autophagy in antigen-specific effector CD8+ T cells, present exciting subjects for future research. Although these studies indicate the ability of autophagy to reprogram CD8+ T cell metabolism, modify epigenetic marks, and limit effector functions, they report different functional consequences regarding tumor control resulting from autophagy activation in T cells. The discrepancies may arise from different functional outcomes observed in long-term (knockout models) versus acute loss of autophagy. Still, these studies collectively support the central role of autophagy in modulating anti-tumor CD8+ T cell responses. However, the precise mechanisms through which autophagy influences T cell memory formation are not yet fully understood. Autophagy likely acts as a double-edged sword in regulating T cell memory formation, with outcomes dependent on physiological and environmental conditions.

During CAR T cell production, metabolic reprogramming occurs when these cells are activated with anti-CD3 and anti-CD28 magnetic beads, transitioning from fatty acid phosphorylation to glycolysis. During this activation process, T cells that require less glucose tend to differentiate into T_mem_ cells, while those requiring more glucose tend to differentiate into T_eff_ cells (62). Furthermore, *in vitro* stimulation of T cells with anti-CD3 and anti-CD28 antibodies induces autophagy (63). Hence, autophagy may modulate the metabolic program of CAR T cells even during CAR T cell manufacturing. The effects of autophagy modulation on CAR T cell metabolism, fate, and function are summarized in **Table 1**.

**Table 1:** Effects of autophagy modulation on (CAR) T cell metabolism, fate, and function.

Autophagy modulation		Effects on (CAR) T cells metabolism	Effects on (CAR) T cell fate and function	References
Autophagy induction	IL15/MTORC1^Weak^ signaling	Promoting mitochondrial biogenesis and FAO	T cell memory formation	(58)
	Carbon monoxide- induced mitophagy	Modify the mitochondrial function and epigenetically reprogram T cells toward a superior antitumor phenotype	Increased antitumor function of T cell	(64)
Autophagy inhibition	*atg5^-/-^* CD8^+^ T cells	Enhanced glucose metabolism	Reduced CD8^+^ T_mem_ formation	(59)
	*Irgm1*-deficient CD4^+^ and CD8^+^ T cells	Increased glucose metabolism and glycolysis	Increased apoptosis; decreased T cell function	(65)
	Mitophagy inhibition	Accumulated depolarized mitochondria	Functional, transcriptomic and epigenetic characteristics of terminally exhausted T cells	(66)
	*Pik3c3*-deficient T cells	Impaired cellular metabolism and reduced levels of active mitochondria upon T cell activation	Failure to differentiate into T helper 1 cells	(61)
	PtdIns3-kinase type III inhibition by 3-methyladenine (3MA) or lysosomal acid hydrolase inhibition by leupeptin and ammonium chloride (L/N)	Inefficient mitochondrial respiration	PTPN1 turnover is reduced, leading to defective TCR-mediated signaling and energy of T cells	(27)
	*Ambra1*-deficient	Perturbed mitochondrial homeostasis	Impaired TCR-mediated cell cycle control; impaired autophagy flux	(49, 67)
	*atg5* or *atg7* deletion	Dysregulated lipid biosynthetic pathways; dysregulated mitochondrial FAO	Survival defects; compromised formation of T_mem_ cells	(29)
	Conditional *atg7* KO mouse model in T cells	Decreased ATP generation	Defective IL2 and IFNG production by T_eff_ cells; reduced proliferation after stimulation	(18)

### 5.2 Metabolic signature governing (CAR) T cell autophagy

**Figure 5:**
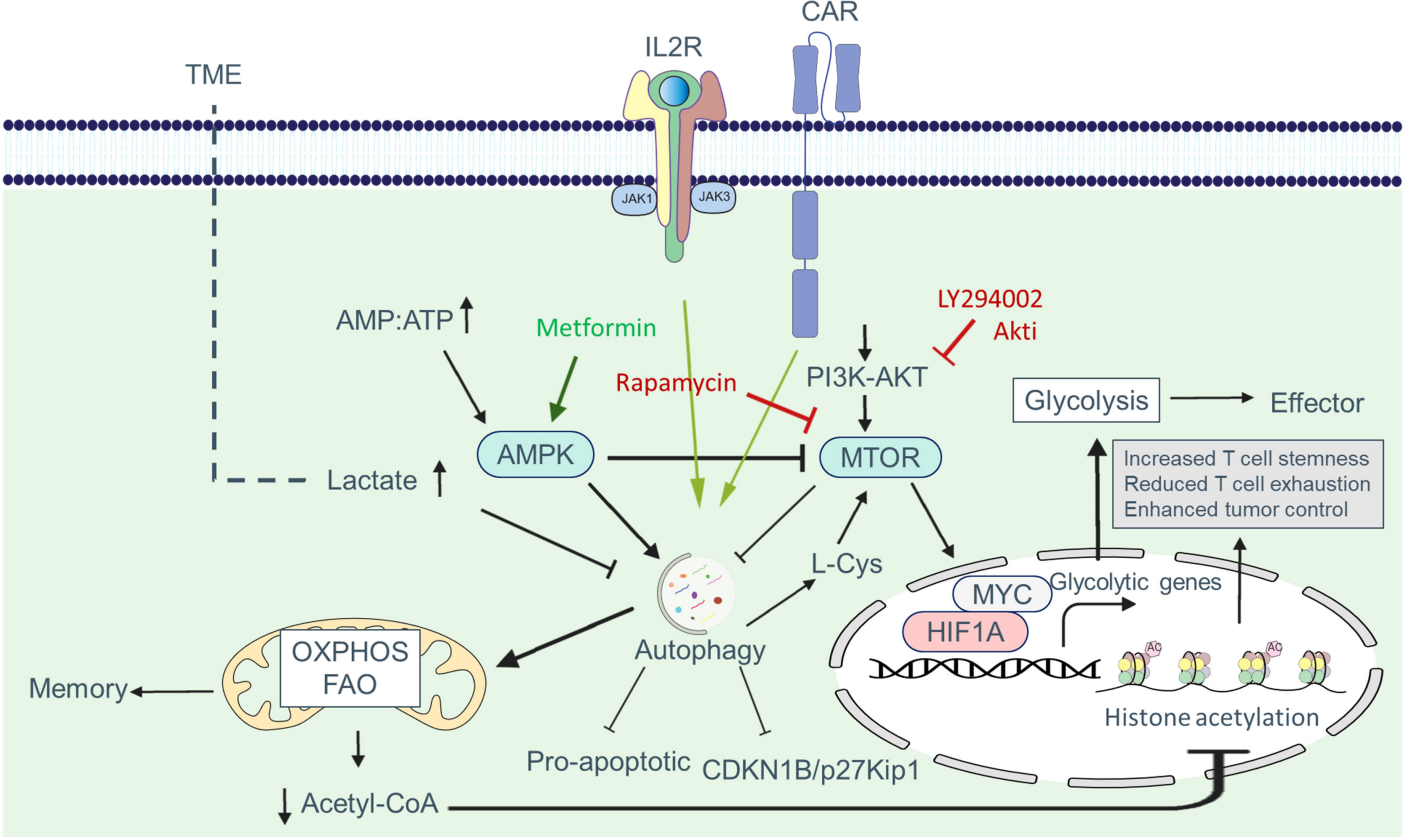
Autophagy-metabolism axis in CAR T cells. Autophagy is triggered in CAR T cells in response to tumor-associated antigens, allowing these cells to better adjust to metabolic demands for proliferation and activation. One of the signaling pathways increased in CAR T cells is the PI3K-AKT-MTOR pathway, which enhances the expression of glycolytic genes such as SLC2A1/GLUT1 via the transcription factors MYC and HIF1A. Increased glycolysis supplies the metabolic mediators required for cell growth and allows cells to develop an effector phenotype. Autophagy can be inhibited in part by MTOR. In the presence of nutrient deficiency in the tumor microenvironment, the AMP : ATP ratio rises, resulting in AMPK activation. AMPK shifts metabolism to OXPHOS and FAO by blocking MTOR and activating autophagy, which produces more ATP and leads to memory formation. Tumor metabolites such as lactate can hamper the adaptation of CAR T cells in the TME by inhibiting autophagy. The reduction of acetyl-CoA following OXPHOS reduces histone acetylation at effector and exhaustion loci, which helps reduce exhaustion, increase stemness and increase the antitumor activity of CAR T cells.

Appropriate regulation of autophagy activity is necessary to control CAR T cell function. Metabolic changes induced by the TME can affect autophagy in CAR T cells. Under nutrient-depleted microenvironments, autophagy promotes metabolic adaptation by facilitating the availability of biomolecules required by CAR T cells (**Figure 5**). Xia et al. demonstrated in a study that tumor-infiltrating T cells have dysfunctional autophagy and reduced levels of RB1CC1/FIP200, a protein essential for autophagosome formation. The decrease in RB1CC1 expression is caused by tumor-derived lactate, which disrupts the balance between pro- and anti-apoptotic factors, enhancing tumor immune evasion (68).

T cells possess nutrient-sensing systems comprising sensors, transporters, and signaling proteins that enable them to detect and respond to fluctuations in nutrient availability. Recent research has focused on understanding how T cells sense nutrients and how alterations in nutrient availability, such as glucose, amino acids, and fatty acids, can affect autophagy in these cells. Potassium overload or nutrient deficiency in the TME limits nutrient uptake by T cells, leading to autophagy via MTORC1 inhibition. Vodnala et al. found that increased potassium levels in the TME reduce nutrient uptake by CD8+ T cells, activating autophagy and promoting metabolic reprogramming towards OXPHOS, which preferentially utilizes acetyl coenzyme A. Reduced acetyl coenzyme A content results in decreased histone acetylation at effector and exhaustion loci, leading to increased T cell stemness, reduced T cell exhaustion, and enhanced tumor control (69). Moreover, autophagy activation in CD8+ T cells within the TME reduces T cell exhaustion and improves tumor control (69). Further studies can provide insights into the role of other tumor microenvironmental factors in altering nutrient sensing in T cells.

Autophagy is crucial for maintaining mitochondrial integrity and is essential for metabolic reprogramming to produce T_eff_ cells. Regulatory T cells (T_reg_ cells) infiltrating tumors express high levels of ARG2 (arginase type II), leading to the degradation of intracellular arginine and decreased MTOR activation by arginine, contributing to high levels of autophagy (70). Loss of autophagy in T_reg_ cells switches metabolic pathways from oxidative phosphorylation to glycolysis with active MTORC1 and MYC, resulting in FOXP3 instability (70, 71). Autophagy-deficient T_reg_ cells are more prone to apoptosis and functional problems (71). Recent studies have shown that PFKFB3 (6-phosphofructo-2-kinase/fructose-2,6-biphosphatase 3) activity increases the rate of glycolysis and is related to the Warburg effect. Knockin and knockout experiments have positioned the glycolytic enzyme PFKFB3 upstream of the autophagy machinery. The metabolic consequence of PFKFB3 deficiency in T cells is the weakening of autophagic activity (72, 73). Therefore, metabolic changes in CAR T cells can also affect autophagic pathways (**Figure 5**). The effects of metabolic modulation on CAR T cell autophagy, fate, and function are summarized in **Table 2**.

**Table 2:** Effects of metabolic modulation on (CAR) T cells autophagy, fate, and function.

Metabolic modulation		Effects on (CAR) T cell autophagy	Effects on the (CAR) T cell fate and function	References
Inhibiting glycolysis during *in vitro* CAR T cell expansion to limit differentiation	2-Deoxy-D-glucose (2DG) (inhibitor of glucose metabolism)	There are no data in T cells, but activation of autophagy in other cells	Inhibited T cell glycolysis; promotes the formation of T_mem_ cells and antitumor function; maintain the stem-like phenotype	(62, 74–76)
	PI3K-AKT-MTOR inhibition	Autophagy induction	Reduce glycolytic activity; increase the percentage of T_n_ and T_cm_, and promote the killing function of CD8^+^ T cells; enhances the CAR-positive expression rate	(77–82)
Selecting the CAR structure that inhibits glycolysis to maintain low differentiation	CD28 (promotes T cell glycolysis by upregulating SLC2A1/GLUT1, PDK1 (pyruvate dehydrogenase kinase, isoenzyme 1), or by activating MTOR)	Macroautophagy induction	More effector memory T cells in CAR T cell subtypes	(44, 83–85)
	TNFRSF9/CD137/4-1BB (Promotes FAO mainly through the STK11/LKB1-AMPK signaling pathway; maintains higher levels of spare respiratory capacity, and mitochondrial biogenesis, and increases OXPHOS	Autophagy inhibition; there are no data in (CAR) T cells	TNFRSF9/4-1BB CAR T cells proliferate slowly but persistently; CAR T cell subtype (T_cm_)	(86–90)
Optimizing media for CAR T cell production	Arginine (promotes OXPHOS and inhibits glycolysis)	Preventing L-arginine depletion induces autophagy	Increased T cell survival and persistence; acquisition of a memory phenotype; improved antitumor response	(91–93)
	IL2 (promotes glycolysis)	Autophagy induction	Rapid T cell proliferation; drives terminal differentiation or activation-induced cell death	(94, 95)
	IL15 (reduces MTORC1 activity and inhibits their glycolytic activity)	Autophagy induction	Preserves the stem cell memory phenotype of CAR T cells	(96, 97)
	IL21	Activates MTORC1 and MTORC2 and suppresses autophagy during T_reg_ differentiation	Shifts metabolism towards FAO and OXPHOS to promote the formation of T_cm_ cells	(98, 99)

## 6 Therapeutic implications of the autophagy-metabolism axis in CAR T cell therapy

### 6.1 Targeting the autophagy-metabolism axis in CAR T cells

#### 6.1.1 CAR construct design

CAR constructs are designed with an extracellular domain comprising an antigen-specific single-chain variable fragment/scFv of an antibody linked to an intracellular signaling domain of a T cell receptor. Synthetic CARs can incorporate co-stimulatory domains that activate different signaling pathways upon antigen recognition (100). Designing a CAR with a co-stimulatory domain that regulates autophagy and metabolism in CAR T cells can influence their function. For example, the CD28 co-stimulatory domain enhances aerobic glycolysis and promotes effector memory differentiation in CAR T cells (86). CD28 stimulation activates the AKT-MTOR signaling pathway and HIF1A transcription factor, leading to increased glycolysis through enhanced glucose uptake and expression of glycolytic enzymes. Additionally, T cells redirected with CD3 and CD28 co-stimulation show upregulation of positive autophagy regulators and downregulation of negative autophagy regulators. Autophagy has been shown to enhance T cell survival by preventing cell death (19, 23). Therefore, CD28 co-stimulation supports highly proliferating and metabolically active CAR T cells.

In contrast to CD28, the TNFRSF9/CD137/4-1BB co-stimulatory domain promotes mitochondrial biogenesis and oxidative metabolism (86). CAR T cells with TNFRSF9 exhibit longer persistence and a higher T_cm_ phenotypes than those with CD28 (86). Thus, TNFRSF9 enhances central memory differentiation and improves the proliferation and persistence of CAR T cells *in vitro*. However, it is important to note that human TNFRSF9 can be degraded through the autophagic pathway (101), which may limit the potential of autophagy induction in CAR T cells containing TNFRSF9. Further studies are needed to elucidate the role of TNFRSF9 in autophagy regulation in CAR T cells.

Fourth-generation CAR T cells are designed to incorporate transgenic cytokines to enhance proliferation and persistence (102). Commonly used cytokines include IL12, IL15, and IL18, which affect T cell metabolism. For instance, IL15-treated CAR T cells exhibit reduced MTORC1 activity, leading to a decrease in glycolysis. Furthermore, IL15-treated CAR T cells express more genes related to FAO and display elevated levels of OXPHOS. These metabolic changes are accompanied by autophagy activation, enhancing CAR T cell anticancer activity *in vivo* by preserving stem cell memory and improving proliferative efficiency (103–105). These observations indicate that CAR constructs with different co-stimulatory domains activate distinct metabolic and autophagy pathways in T cells, thereby influencing their fitness within the TME (106).

#### 6.1.2 Modulation of signaling pathways

Targeting components of the autophagy-metabolism axis using specific compounds is a promising approach to modulate CAR T cell biology and improve their antitumor potential. Studies have shown that the PI3K-AKT-MTOR pathway promotes the differentiation of terminal effector T cells rather than T_cm_ cells, reducing CAR T cell persistence (107). To address this, researchers utilize inhibitors targeting the glycolysis pathway, PI3K, AKT, and MTOR to decrease T cell glycolysis levels ex vivo, resulting in a more functional T cell product *in vivo* (108). Recent reports have highlighted the essential role of T cell-intrinsic mitochondrial regulation by autophagy in sustaining immunity against tumors (66). Inhibiting the PI3K-AKT-MTOR pathway can enhance autophagy, a well-described homeostatic process that promotes T cell memory and mitochondrial fitness (29, 109). The effects of modulating autophagy-metabolism signaling pathways on CAR T cells are summarized in **Table 3**.

**Table 3:** Effects of autophagy-metabolism signaling pathway modulation on (CAR) T cells.

Signaling pathway modulation		Outcome on (CAR) T cell	References
PI3K	LY294002 IC87114	Increasing T_n_ and T_cm_ populations of CD33 CAR T *in vivo*; improving CAR T cell persistence and reducing tumor burden *in vivo*	(108)
AKT	A-443654	Rescuing short-lived effector cells from deletion due to sustained AKT activation; enhancing P14 CD8 effector memory T cells *in vivo*	(110)
	Akti-1/2	Preventing CAR T cell differentiation; increasing cytokine production and cytotoxicity; exhibiting greater antitumor efficacy and expansion *in vivo*	(111)
MTOR	Rapamycin	Reduced expression of glycolytic enzymes and improved mitochondrial fitness; less-differentiated stem cell-like memory (T_scm_) phenotype; reduced expression of exhaustion markers; higher antiapoptotic properties; increased proliferative capacity	(96, 112)
	*mtor* deletion	Ameliorates CD4^+^ T cell apoptosis during sepsis by improving autophagosome-lysosome fusion	(113)
	Cotreatment with MTOR inhibitors during IL2-mediated *ex vivo* expansion	Upregulated CXCR4 and bone marrow migration and AML elimination by CAR T cells	(114)
	Aptamer-targeted siRNA inhibition of MTORC1	Enhanced differentiation into T_mem_ cells; enhanced antitumor immunity	(115)
AMPK	Metformin	Inhibits proliferation and cytotoxicity; induces apoptosis	(116)
	T cell-specific deletion of *Prkaa1/AMPKα1*	Reduced mitochondrial bioenergetics and cellular ATP in response to glucose limitation	(117)

#### 6.1.3 Genetic manipulation

Genetic modifications targeting the autophagy-metabolism axis can enhance the antitumoral function of CAR T cells. Studies have shown that in bone marrow chimeras with ATG5-deficient donor cells, there is a significant increase in IFN-γ and TNFα-producing CD8+ T cells in tumor-infiltrating lymphocytes, leading to improved tumor control (59). Similarly, CD8+ T cells lacking ATG5 display an effector memory phenotype and produce more IFN-γ and TNFα (118). Autophagy-competent mice receiving subtherapeutic doses of *atg5-/-* T cells also demonstrate controlled tumor growth (118). Therefore, genetic ablation of Atg5 can increase the metabolic activity and tumor-killing ability of CAR T cells. Consequently, Atg5 ablation provides a novel therapeutic opportunity to improve the efficacy of CAR T cell immunotherapy in solid tumors. Researchers have also designed CRISPR-Cas9 gene editing to knock out the autophagy function in CAR T cells as a novel immunotherapy approach to treat ovarian cancer (119).

## 7 Targeting the autophagy-metabolism axis as a CAR T cell combination therapy

### 7.1 Improving CAR T cell trafficking

The TME is known to impede the infiltration and function of CAR T cells in solid tumors. Autophagy has been shown to regulate the expression of chemokines in tumor cells that mediate immune cell migration to the tumor (120, 121). Inhibiting autophagy in tumors may modify the TME and promote the production of TH1-type chemokines, facilitating the trafficking of CAR T cells (120, 122). A study in lung tumor models demonstrated that simultaneous treatment with chemotherapy and MEK inhibitors promotes mitophagy and recognition of mitochondrial DNA by TLR9 (120, 121). TLR9 signaling leads to the expression of CXCL10 in tumor cells and the recruitment of CD8+ T cells to the tumors (121). Therefore, targeting autophagy in tumor cells can be utilized to increase the migration of T cells to the tumor and improve the antitumor response in combination with immunotherapies such as immune checkpoint blockade (ICB) or CAR T cell therapy.

### 7.2 Lactate

Increasing lactate levels in the TME can inhibit autophagy in CAR T cells (68). Therefore, the use of lactate dehydrogenase (LDH) and monocarboxylate transporter (MCT) inhibitors (123) in combination with CAR T cell therapy may enhance autophagy, thereby improving the survival and proliferation of CAR T cells.

### 7.3 Immune checkpoint blockade

CD274/PD-L1 molecules are overexpressed in several types of cancer (124). The interaction between CD274/PD-L1 and PDCD1/PD1 on T cells prevents cytotoxic T cell activation, allowing cancer cells to evade the immune system (124). The CD274/PD-L1/PD1 axis is critical in regulating T cell metabolism. PDCD1/PD1 blocks glycolysis by inhibiting the PI3K-AKT-MTOR pathways and downregulating SLC2A1/GLUT1 expression, which is crucial for T cell activation (125). Additionally, PDCD1/PD1 activates AMPK, a kinase that regulates fatty acid oxidation and induces autophagy by activating ULK1 (126). In contrast, CD274/PD-L1 induces glycolysis in tumor cells, depleting glucose from the TME (127), which is essential for CAR T cell activity. Therefore, inhibitors targeting the PDCD1/PD1-CD274/PD-L1 axis can combined with CAR T cells to affect the autophagy-metabolism pathway, thereby improving the antitumor function of CAR T cells.

### 7.4 Metabolic reprogramming

Metabolic reprogramming of the tumor microenvironment can significantly impact CAR T cell function. One approach to modulating the TME is to target specific metabolic pathways in tumor cells. For example, glycolysis or glutamine metabolism inhibitors can alter the metabolic landscape of the TME, potentially enhancing CAR T cell efficacy (33, 128). Furthermore, the manipulation of specific metabolic checkpoints, such as the inhibition of key enzymes involved in lipid metabolism or amino acid utilization, may provide opportunities to enhance CAR T cell function within the TME (129, 130). Strategies aimed at metabolic reprogramming of both the TME and CAR T cells hold promise for improving the therapeutic outcomes of CAR T cell combination therapy.

### 7.5 Hypoxia and autophagy

Hypoxia, a condition of reduced oxygen availability, is a common feature of the TME and has been associated with immunosuppression and resistance to therapy. Autophagy plays a crucial role in cellular adaptation to hypoxic stress, including the survival of tumor cells. Targeting the interplay between hypoxia and autophagy within the TME presents a potential strategy to enhance CAR T cell therapy. Modulating hypoxia-inducible factors (HIFs), which regulate both metabolism and autophagy, can influence the function of CAR T cells in hypoxic tumor regions (131, 132). Combination therapies that target hypoxia, such as hypoxia-activated prodrugs or inhibitors of HIF signaling, in conjunction with autophagy modulation, may promote CAR T cell persistence and antitumor activity.

## 8 Conclusions

This review investigates the autophagy-metabolism axis in (CAR) T cells. Autophagy is present in naïve T cells at a basal level; however, upon TCR stimulation, autophagy increases to support the metabolic demands for activation and proliferation. Various aspects of (CAR) T cell performance against the tumor, including survival, proliferation, phenotype, and exhaustion are affected by the interaction of autophagy and metabolism. In general, inhibition of autophagy in (CAR) T cells leads to increased glycolysis, inefficient mitochondrial respiration, reduced ATP production, and decreased FAO. These metabolic changes contribute to an effector phenotype. However, inhibition of autophagy may increase apoptotic proteins and cell cycle inhibitors that compromise the survival and proliferation of (CAR) T cells.

Conversely, the induction of autophagy is associated with increased OXPHOS, and FAO, which helps (CAR) T cells to differentiate toward the memory phenotype. Also, activation of autophagy can lead to increased survival and proliferation of (CAR) T cells by destroying pro-apoptotic proteins and cell cycle inhibitors. Nutrient-deficient conditions in the tumor microenvironment can cause metabolic changes in CAR T cells. The metabolic stress caused by the TME impairs effector T cell function and induces apoptosis. An elevated level of autophagy in CAR T cells may boost T cell fitness and survival in the TME; therefore, enhancing autophagy in CAR T cells before transfusion to patients might improve the effectiveness of the therapy (133). Furthermore, blocking autophagy in tumors may improve CAR T therapy efficacy by increasing tumor-associated antigen expression. Additionally, the metabolites produced by tumor cells can modulate autophagy in CAR T cells by influencing the components of the autophagy pathways. However, despite recent progress in this area, many aspects concerning autophagy, metabolism, and CAR T cell function remain unknown. Further studies are warranted to gain a comprehensive understanding of this axis in CAR T cells, ultimately identifying targets that can be manipulated to enhance the antitumor function of these cells. Continued research in this field has the potential to advance the development of more effective CAR T cell therapies.

## Author contributions

HM conceived the idea. AP, BA and TS wrote the first draft of the manuscript and drew the figures. JH, DK, BB, and HM gave critical comments and revised the manuscript. All authors read and approved the final version of this work.
